# The Relationship Between Parental Attachment and Mobile Phone Dependence Among Chinese Rural Adolescents: The Role of Alexithymia and Mindfulness

**DOI:** 10.3389/fpsyg.2019.00598

**Published:** 2019-03-20

**Authors:** Xiaoqing Li, Chenrui Hao

**Affiliations:** ^1^College of Psychology and Sociology, Shenzhen University, Shenzhen, China; ^2^Shenzhen Key Laboratory of Affective and Social Cognitive Science, Shenzhen University, Shenzhen, China

**Keywords:** parental attachment, mobile phone dependence, alexithymia, mindfulness, adolescents

## Abstract

Mobile phone has experienced a significant increase in popularity among adolescents in recent years. Findings indicate dependence on mobile phone is related to poor parent-child relationship. However, previous research on mobile phone dependence (MPD) is scant and mainly focus on adult samples. In this view, the present study investigated the association between parental attachment and MPD as well as its influence mechanism, in sample of adolescents in rural China. Data were collected from three middle schools in rural areas of Jiangxi and Hubei Province (*N* = 693, 46.46% female, *M*_age_ = 14.88, *SD* = 1.77). Participants completed the Inventory of Parent and Peer Attachment (IPPA), the twenty-item Toronto alexithymia scale (TAS-20), the Mindful Attention Awareness Scale (MAAS) and the Mobile Phone Addiction Index Scale (MPAI). Among the results, parental attachment negatively predicted MPD and alexithymia were exerting partial mediation effect between parental attachment and MPD. Further, mindfulness acted as moderator of the relationship between alexithymia and MPD: The negative impact of alexithymia on MPD was weakened under the condition of high level of mindfulness. Knowledge of this mechanism could be useful for understanding adolescents’ MPD in terms of the interaction of multiple factors.

## Introduction

Mobile phone dependence (MPD) refers to an excessive indulgence to activities related to mobile phones, accompanied by eagerness and a strong and continuous dependence on mobile phones, which results in loss of self-control and compromised psychological and social functioning among individuals (Yen et al., 2009; Seo et al., 2016; Liu et al., 2018). The prevalence rate of MPD has been estimated to be approximately 15–40% among adolescents in many countries, and the rate is still in fast growth (e.g., Leung, 2008; Xu et al., 2012; Nikhita et al., 2015; Jun, 2016). MPD in adolescence result from a complex interplay of multiple individual and social risk factors (Billieux, 2012; Choliz, 2012). However, the present studies mainly focus on mobile phone use from the perspective of individuals. For example, research found that individual factors, such as personality (Roberts et al., 2015), self-esteem (Kim and Sun, 2016; Kim and Koh, 2018), impulsivity (Leung, 2008; Billieux et al., 2010), as well as poor self-regulation skills (Gökçearslan et al., 2016; Mei et al., 2017), were associated with MPD. More research is needed to provide empirical evidence on the special contributions and the interplay of the various internal and external factors, so that a more integrated understanding about MPD in adolescence can be represented.

### Parental Attachment and MPD

Attachment refers to a deep and lasting emotional connection established by an individual in the interaction with significant others, expressing the individual’s tendency to seek closeness or contact with specific objects (Bowlby, 1969, 1973). According to attachment theory, addiction or dependence behavior is, to some extent, the transfer and compensation of individual attachment needs (Flores, 2006). Bowlby (1988) assumed attachment is active over the entire life span and it is manifested in thoughts and behaviors related to seeking proximity to attachment figure in times of threat or need. When individuals with difficulties in developing intimate relationships face the painful or stressful events, they often use emotional connections with material or behavior to compensate for the attachment needs that cannot be obtained from their significant others (Flores, 2001). For example, Kim and Koh (2018) found that college students with high avoidant attachment used their phones to distance themselves from negative relationships.

Mobile phone represents a relationship-maintaining tool and a store of social connections and memories, this makes it much easier to become a target of compensatory attachment than other material objects (Konok et al., 2016). Results from studies on young adolescents and college students show that insecure attachment leads to serious MPD (e.g., Lepp et al., 2016; Kim and Koh, 2018). However, the existing studies on the link between attachment and MPD, a new form of dependent behavior emerged in recent years, is still insufficient. Thus, the current study attempts to analyze the relationship between parental attachment and MPD as well as the influencing mechanism systematically, such as the mediation process (i.e., how attachment is linked to MPD) and moderation process (i.e., when attachment is linked to MPD).

### Mediator and Moderator of the Relationship Between Parental Attachment and MPD

#### Alexithymia as a Mediator

Alexithymia is characterized by reduced capacity to identify, analyze and express emotions, restricted imagination, and an externally oriented thinking (Timoney and Holder, 2013). Its development is an accumulated process that starts in childhood and continues to strengthen through social environment (Kauhanen et al., 1993). And the individual differences in children’s emotion cognition and understanding partly derive from parents’ efforts to model and explain emotions (Brownell et al., 2013). As a result, alexithymia is greatly influenced by individual life experiences, particularly the relationship with attachment figures such as parents. The insecure attachment will inhibit individuals’ understanding of emotions and negatively influence their ability to identify and describe their feelings, so that these insecurely attached people are prone to have more severe alexithymia (Thorberg et al., 2011).

For those with severe alexithymia, emotional defects make it difficult to express their feelings and to understand others in face-to-face communication. Therefore, it is more likely for them to experience failure in communication as well as the establishment and maintenance of relationships (Rieffe et al., 2006; Heaven et al., 2010; Gao et al., 2017). Lack of acceptance and sense of belonging may urge the individuals to use other ways to meet their needs. Since there is no physical presence and proximity to others and no direct observation of others through online activities, mobile phones provide a more convenient and favored form of communication medium to better express emotions and to fulfill their unmet social needs (Scimeca et al., 2014; Xu et al., 2016). Based on this, alexithymia is hypothesized to positively predict MPD severity, and parental attachment is assumed to affect MPD through alexithymia.

#### Mindfulness as a Moderator

As a dispositional trait, mindfulness is considered a conscious attention and awareness of external and internal stimuli in the present moment (Brown and Ryan, 2003). Ryan and Deci (2000) proposed that mindfulness serves as an important mechanism in allowing individuals to disengage from automatic thoughts and unhealthy behavior patterns, while simultaneously promoting informed and self-endorsed behavior regulation. Researches on the impact of mindfulness have shown promising results in the areas of reduction of psychological distress and affective disturbance, as well as increase in affect/emotion regulation and maintenance of emotional stability (Creswell et al., 2007; Brown et al., 2009; Goldin and Gross, 2010; Eichel and Stahl, 2017). In recent years, mindfulness has also been found to have positive effects on substance abuse (Brewer et al., 2009; Britton et al., 2010; Bowen et al., 2017). Hence, mindfulness is increasingly viewed as a factor in making human function more optimal.

##### Mindfulness and attachment

The increasing level of mindfulness leads to a potential reduction in behaviors that are seen as the characteristic of attachment insecurity, such as experiential avoidance (Goodall et al., 2012). For those mindful individuals, who are open and acceptant to their life experiences, they often strive to experience each moment at a deep level with non-judging (Brown and Ryan, 2003). And in the context of the activation of the attachment system, mindful awareness of each moment helps individuals to experience potential threats (i.e., parent’s insensitivity) by enabling them to accept these behaviors without judgment and continue to engage in the current moment (Mikulincer and Shaver, 2007; Saavedra et al., 2010). Mindfulness, therefore, prevents attachment system of individuals from being activated by fundamentally altering the perception of negative behaviors, so that they are not automatically seen as a threat to relationships. And this is likely to reduce the possibility for the people who have unavailable attachment figure to seek out some undesirable attachment alternatives (e.g., substance, game, and/or internet). Therefore, we hypothesize that the mindful individuals can modulate the negative effects brought by unavailable or insensitive attachment figure.

##### Mindfulness and alexithymia

Mindfulness has been linked to emotional processing (Hayes and Feldman, 2004). Carmody and Baer (2008) have reported that mindfulness can improve subjective well-being and life quality by increasing individual positive emotional experience. One of the keys may be that mindfulness is associated with greater emotion differentiation and less emotional disorder (Hill and Updegraff, 2012). While these difficulties on identifying and describing feelings are the most typical issues in alexithymic individuals. And in contrast to alexithymia, mindfulness encourages individual to hold curiosity and attentiveness to their inner experiences and to become familiar with the thoughts or feelings appeared in the body (Gilbert et al., 2012). Thus, mindfulness could be an appropriate factor for buffering the deficiency and a series of negative effects brought by alexithymia, and thus these people are less likely to have severe dependence on mobile phone.

### The Chinese Rural Adolescents

There are regional, particularly urban-rural, differences in social and economic development in China (Chen et al., 2010). Despite dramatic social and economic changes in urban areas, the rural areas are less developed (Chen and Santo, 2016). This drives a large number of rural labors into the city. As a result, the social support of the families in rural areas has been weakened and the family structure becomes unstable (Lv and Liu, 2011). Over time it is detrimental to the emotional well-being and social functioning of rural adolescents, and makes them more likely to have problem behaviors such as substance abuse (Shek, 2002, 2005; Wang and Mesman, 2015). Especially for those who are left-behind, the long-term weakened parent-child bonding and communication presumably bring a further adverse impact on the adolescents. Thus, the dysfunction of attachment may make the rural group more dependent on their mobile phones. In addition, rural adolescents account for 48.76 percent of the total in China (National Bureau of Statistics of China, 2011). But the attention paid to the rural group is insufficient in the research of mobile phone use. Against the background above, by focusing on a Chinese rural sample, we explore the impact of possible deficiencies in their parental attachment on MPD.

### The Present Study

**FIGURE 1:**
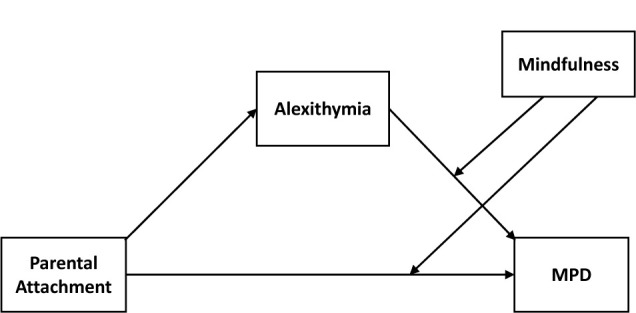
The hypothetical structure model. MPD, mobile phone dependence.

The objectives of the present study were twofold. Firstly, the present study aimed to examine the influence of parental attachment on MPD as well as the mediating role of alexithymia in the association in Chinese rural adolescents. We expected that parental attachment could not only negatively predict MPD but also influence it through the mediating effect of alexithymia in rural adolescents. Secondly, this study would also explore the mindfulness as a potential protective factor for MPD. We hypothesized that adolescents who displayed higher (vs. lower) levels of mindfulness would be less likely to engage in MPD. The hypothetical structure model was shown in Figure 1.

## Materials and Methods

### Participants

Participants were recruited from three middle schools in rural areas of Hubei and Jiangxi Province, central China. We chose these regions specifically for the majority of the population is classified as peasants and its high level of out-migration of laborer to big cities and the east areas (National Bureau of Statistics of China, 2017). A total of 829 students were approached, and 693 valid questionnaires were recovered (136 participants were excluded for the missing data on the main variables). Among the data, there were 352 boys and 322 girls, with an average age of 14.88 ± 1.77. The proportion of boys and girls in each grade were basically matched.

Alongside consent from the school principal and concerned teachers, full written parental consent was obtained for all participating students. Furthermore, the research design was approved in an ethical review process conducted by the Human Research Ethics Committee of Shenzhen University. This study took the respondents approximately 30 min to complete all the questionnaires. During this process, all invited participants were voluntary and guaranteed confidentiality.

### Measures

#### Parental Attachment

The Inventory of Parent and Peer Attachment (IPPA; Armsden and Greenberg, 1987) is used to measure the intimate relations between adolescents and their family and peers. For the purpose of this study, only the parental attachment subscale was selected. Since there were some left-behind children in the subjects whose caregivers might not be their parents (one or both of their parents went out to work), the “*father or mother*” in the question was revised to “*parent or guardian (the person who mainly takes care of you in life)*”. It consists of three factors: trust (e.g., “*My parent or guardian respects my feeling*”), communication (e.g., “*My parent or guardian senses when I’m upset about something*”) and alienation (e.g., “*I feel angry with* *my parent or guardian*”), with 25 items in all. The five-point Likert scale requires the examinee to circle a selected number on the range 1 (almost never true) to 5 (almost always true). The total parental attachment scale reflects general parental attachment security, with lower scores indicating more insecure parental attachment as reflected in lower trust and communication to parents, and higher sense of alienation. In this study, the *Cronbach’s*α was 0.71.

#### Alexithymia

The twenty-item Toronto alexithymia scale (TAS-20; Bagby et al., 1994) consists of three factors: difficulty identifying feelings (DIF), difficulty describing feelings (DDF), and externally-oriented thinking (EOT), with 20 items in all. The TAS-20 Chinese version had good reliability and validity in the Chinese youth group (Ling et al., 2009). Due to cultural differences, the reliability and validity of EOT dimensions (compared with DIF and DDF dimensions) are relatively poor (Taylor et al., 2003; Zhu et al., 2007; Ling et al., 2009). For this, the present study referred to the previous literature (e.g., Liang et al., 2015), only DDF (e.g., “*I am often confused about what emotion I am feeling*”) and DIF (e.g., “*It is difficult for me to find the right words for my feelings*”), a total of 12 items, were used to represent the degree of alexithymia. It uses a five-point Likert scale (1 = not true, 5 = true), with higher score representing more serious alexithymia. In this study, the *Cronbach’s*α was 0.88.

#### Mindfulness

The Mindful Attention Awareness Scale (MAAS; Brown and Ryan, 2003) is a 15-item single-dimension measure of trait mindfulness. The MAAS measures the frequency of open and receptive attention to and awareness of ongoing events and experience. In order to control for social desirability, participants are instructed to respond to the MAAS in a way that reflects their actual experience rather than in a way they think their experience should be. It uses a six-point Likert scale (1 = almost always, 6 = almost never), with higher score indicating greater degree of mindfulness. Example items include “*I find it difficult to stay focused on what’s happening in the present” and “I could be experiencing some emotion and not be conscious of it until some time later”*. In this study, the *Cronbach’s*α was 0.87.

#### Mobile Phone Dependence

The Mobile Phone Addiction Index Scale (MPAI; Leung, 2008) contains four factorial components: control carving (e.g., “*Someone said I spent too much time on my phone*”), the feeling anxious and lost (e.g., “*If I haven’t turned on my phone or checked my phone for a while, I will get edgy and restless*”), the withdrawal (e.g., “*When I felt lonely, I used to chat with others on my mobile phone*”), and the productivity loss (e.g., “*The time I spend on my phone directly leads to my low efficiency, such as learning*”), with 17 items in all. It uses a five-point Likert scale (1 = not at all, 5 = always), with higher score indicate a higher degree of individual’s MPD. In this study, the *Cronbach’s* α was 0.91.

#### Demographic Variables

Sociodemographic information was also measured. The information included the following: (1) basic demographic information: gender, age, grade, etc., (2) mobile phone use: hours of use, main activities, etc.

### Data Analysis

The participants were investigated in group of classes. SPSS23.0 was used for data analysis, including descriptive statistics, reliability analysis and correlation analysis among variables; and the macro program PROCESS of SPSS (Hayes, 2013) was employed for structural equation modeling and the moderated mediation effect analysis. Preliminary analysis found that there is only 0.03 and 0.06% missing data in gender and age, respectively. However, due to the small proportion of missing data and the particularity of age, no special treatment was carried out for the missing data. Subjects with missing data were excluded when dealing with data related to gender and age.

Harman’s single-factor test was used to examine the issue of common method variance before processing data. The basic assumption of this technique was that if a substantial amount of common method variance is present, either (a) a single factor will emerge from the factor analysis or (b) one general factor will account for the majority (>40%) of the covariance among the measures (Podsakoff et al., 2003). In this study, the first factor accounted for 14.74% of the covariance among all the items, suggesting that there was no severe common method variance.

## Results

### Descriptive Statistics and Correlation Coefficients for Each Variable

The mean value, standard deviation and correlation matrix for each variable were listed as followed (Table 1). The correlation analysis showed that MPD was negatively correlated with parental attachment and mindfulness, and positively associated with alexithymia. It indicates that when the adolescents had poorer parental attachment and weaker mindfulness, they were more likely to have severe alexithymia and MPD.

**Table 1:** The mean value, standard deviation, and correlation matrix for each variable (*N*=693).

	*M*±*SD*	1	2	3	4	5	6
Gender	0.48±0.50	1.00					
Age	13.64±1.04	0.01	1.00				
Parental attachment	1.91±0.65	0.01	0.05	1.00			
Alexithymia	2.91±0.49	0.04	0.02	–0.41**	1.00		
Mindfulness	3.83±0.90	0.02	–0.07	0.35**	–0.60**	1.00	
MPD	2.57±0.82	–0.14**	0.14**	–0.29**	0.38**	–0.43**	1.00


*^∗^p < 0.05, ^∗∗^*p* < 0.01; gender is a dummy variable, boy = 0, girl = 1, the average meant the proportion of girls; MPD, mobile phone dependence.*

### The Moderated Mediation Model

According to Wen and Ye (2014), the association between parental attachment and MPD, the mediation of alexithymia in the association and the moderated effect of mindfulness were analyzed, respectively. All variables were standardized, and the PROCESS was used to test the moderated mediation model after controlling for gender and age (Table 2).

**Table 2:** The moderated mediation model (*N*=693).

Outcome Variable	Predictive Variable	*R*	*R* ^2^	*F*	β	95% CI	*t*
Alexithymia	Parental Attachment	0.42	0.18	45.87***	–0.42	[–0.49, –0.35]	–11.65***
	Gender				0.07	[–0.07, 0.21]	1.04
	Age				0.04	[–0.02, 0.12]	1.39
MPD	Parental Attachment	0.51	0.26	32.15***	–0.11	[–0.18, –0.03]	–2.83**
	Alexithymia				0.16	[0.08, 0.25]	3.70***
	Mindfulness				–0.29	[–0.38, –0.21]	–6.81***
	Alexithymia × Mindfulness				0.08	[0.01, 0.15]	2.33**
	Parental Attachment × Mindfulness				–0.02	[–0.09, 0.04]	–0.70
	Gender				–0.29	[–0.42, –0.15]	–4.15***
	Age				0.12	[0.05, 0.18]	3.59***


*^∗^p < 0.05, ^∗∗^*p* < 0.01, ^∗∗∗^*p* < 0.001; Bootstrap sample size = 5000, Bootstrap 95% CI (confidence interval) does not contain 0 value, indicating significant coefficient.*

Expectedly, results showed that parental attachment was significantly associated with both alexithymia (β = -0.42, *t* = -11.65, 95% CI (confidence interval) was [-0.49, -0.35], *p* < 0.001) and MPD (β = -0.10, *t* = -2.76, 95% CI was [-0.18, -0.03], *p* < 0.01). Furthermore, alexithymia showed a significant effect on MPD (β = 0.16,*t* = 3.75, 95% CI was [0.08, 0.25], *p* < 0.001). And the interaction effect between alexithymia and mindfulness on MPD was also found (β = 0.09, *t* = 3.00, 95% CI was [0.03, 0.15], *p* < 0.01).

**FIGURE 2:**
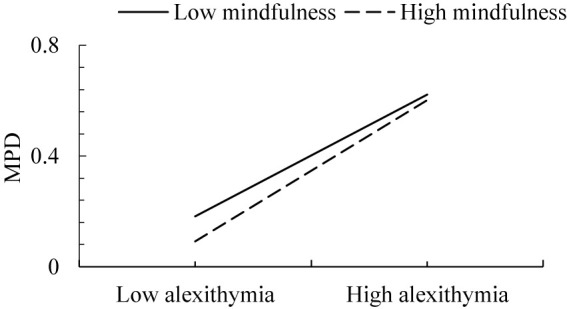
The association between alexithymia and MPD as a function of mindfulness level (low vs. high).

Based on the above results, the moderated mediation model proposed in this study was supported (Wen and Ye, 2014). The direct effect of parental attachment on MPD was significant, and alexithymia played a partial mediating role in the association. Since only the latter half of the mediating pathway of alexithymia was moderated by mindfulness. To further clarify the nature of the moderating effect, all participants were divided into two groups, including the high mindfulness group (1 *SD* above the mean) and the low mindfulness group (1 *SD* below the mean). The significant association was observed. Specifically, mindfulness moderated the associations between parental attachment and MPD. The mediational effect of alexithymia and its 95% CI were shown in Table 3. To better understand the moderating effect of mindfulness, the plot of the relation between alexithymia and MPD at two levels of mindfulness (low vs. high) was demonstrated in Figure 2. As can be seen from Figure 2, the effect of alexithymia on MPD was exacerbated for individuals with lower level of mindfulness in compared with those with higher level of mindfulness.

**Table 3:** The direct effect of parental attachment and mediating effect of alexithymia at two levels of mindfulness.

The levels of mindfulness	Effect Size		95% CI
*M* – *SD*	direct effect	–0.08	[–0.18, 0.01]
	indirect effect	–0.03	[–0.09, –0.02]
*M*	direct effect	–0.11	[–0.18, –0.03]
	indirect effect	–0.07	[–0.11, –0.03]
*M* + *SD*	direct effect	–0.13	[–0.24, –0.02]
	indirect effect	–1.10	[–0.15, –0.06]


## Discussion

This research aimed at testing the relationship between parental attachment and MPD as well as the mediating role of alexithymia and the moderating role of mindfulness in these associations in a sample of Chinese rural adolescents. In support of the hypotheses, parental attachment was associated with MPD. Moreover, alexithymia played the partial mediating role in this association and mindfulness moderated the latter half of the mediating effect of alexithymia. These findings provided a more nuanced picture regarding the association between parental attachment and adolescent MPD, which would provide significant implications for MPD prevention and intervention.

As expected, results demonstrated that there was a negative impact of insecure parental attachment on MPD in rural adolescents. The result was also observed in our previous study, which attachment (avoidance and anxiety) negatively predicted MPD in urban areas (Li and Hao, unpublished). Detailed comparisons cannot be made due to differences in research purposes. Roughly speaking, both studies highlight the stable influence of the attachment between parent and children in the development of MPD, irrespective of rural group or urban group. And then, we further analyzed the content of mobile phone use, and found that most teenagers with insecure attachment used mobile phone primarily for socializing and communicating (e.g., using WeChat or QQ to keep in touch with others). These findings indicated that mobile phone could function as a compensatory attachment target providing a sense of security and substituting for the person’s social connections (Konok et al., 2016). It also implies that attachment theory could help to understand the emerging phenomenon of MPD in recent years, in addition to substance use (e.g., Fletcher et al., 2015) and Internet addiction (e.g., Bolat et al., 2017).

Is MPD an addiction or dependence? An increasing number of researchers suggest that the consequences caused by the excessive use of mobile phone is not exactly the same as addiction (e.g., Billieux et al., 2015). It is not enough to study this issue only from the addiction framework, but also to view mobile phone use in a context that considers the compensatory functions of the device (see Panova and Carbonell, 2018 for more comprehensive review). Some studies have started to focus on “Attachment to Smartphones” to explore the influence of the non-human attachment (Konok et al., 2016; Bodford et al., 2017). Mobile phone has become increasingly indispensable in everyday life due to a desire to maintain a sense of interpersonal connection even, or especially, when devoid of human contact (Bodford et al., 2017). For now, it is not only a simple tool, but also an attachment object for individuals, which is endowed by the more advanced on notation due to the extensive participation of phone users (Konok et al., 2017). In future research, attention should be paid to how to make better use of the multifunctional mobile phones, in particular, its role in satisfying individual emotional and attached needs, as mentioned above.

In addition, the present study found that alexithymia had a strong mediating effect on the association between parental attachment and MPD in rural adolescents. Moreover, the predictive coefficient of parental attachment for alexithymia was higher than that for MPD. According to attachment theory, the function of attachment was mainly to manage interpersonal emotional experience (Sroufe and Waters, 1977). And the destruction of attachment insecurity was achieved through the damage of individual emotional function and then acting on their behaviors (Thorberg et al., 2011). It is precisely because of their negative self-others cognition due to poor parental attachment (Crittenden and Ainsworth, 1989), the emotional ability of alexithymic people, such as emotion identification and expression, was inhibited to some extent. As a result, they were more likely to be rejected by peer than their counterparts (Miller et al., 2005; Heinze et al., 2015).

So how would they meet their interpersonal needs? Chan and Cheng (2016) pointed out that social support obtained through online might be better than through face-to-face interactions. Mobile phone became a tool for the individuals with alexithymia, as similar with the motivation of “compensated use” for Internet addiction, to avoid negative emotions and impacts, as well as to escape from real-world problems (Kardefelt-winther, 2014). Hence, consistent with previous researches (Elhai et al., 2017; Gao et al., 2017; Rozgonjuk et al., 2018), the emotional problem was an important predictor of MPD in rural area. It was particularly important to break the communication barriers caused by improper expression and understanding of emotions.

The other goal of the present study was to seek the factor which can alleviate the negative effects of undesirable attachment on the individual’s dependent behavior. The results indicated that mindfulness significantly moderated the relation between alexithymia and MPD, but not in the relation between parental attachment and MPD. In other words, mindfulness could relieve MPD by regulating alexithymia, although the effect size was relatively small. Consistent with the previous studies, mindfulness played a protective role in the influence of negative factors on individual physical and mental health (e.g., Bajaj et al., 2016; Davis et al., 2016). In particular, mindfulness presumably works by improving an individual’s emotional state and ability (Wang and Huang, 2011). This was why the moderating effect of mindfulness was not significant in the association between parental attachment and MPD. Research evidences suggested that the relation between the two variables worked primarily through emotion-related abilities (Goodall et al., 2012; Pepping et al., 2013). According to Shapiro and colleagues, mindfulness helped individuals to experience internal and external stimuli more objectively, and to reduce the subjective feelings towards undesirable stimuli (Shapiro et al., 2006). Hence, individuals could be more tolerated internal and external experiences, so as to improve negative emotions and enhance positive emotions to maintain physical and mental health (Stevenson et al., 2017). The clear recognition of internal emotional states might be an important role of mindfulness in alleviating the characteristics of alexithymia, thus relieving the negative impact of alexithymia on MPD.

The above result with respect to the mindfulness is promising for the intervention of MPD, especially for those with deficit in emotion area such as alexithymia. Some studies demonstrated the long-term benefits of mindfulness-based interventions with a significant increase in trait mindfulness, especially in improving mental and emotional health functioning (Hofmann et al., 2010; Shapiro et al., 2011; Paul et al., 2013). Promoting the development of emotional management and regulation by improving one’s mindfulness is worth exploring focus of MPD intervention in future research. Some researchers proposed that mindfulness-based interventions were the promising method to prevent and reduce problematic Internet use through preventing adolescents from using the Internet for mood regulation (e.g., Calvete et al., 2017). Similarly, using mindfulness training to alleviate the emotional expression and understanding of alexithymic individuals may reduce their dependence on the online social activities through mobile phones.

## Limitations and Prospectives

Some limitations of this study must be acknowledged. The first limitation is about the research methods: (1) The current study is the cross-sectional design. Because attachment in adolescence is dynamic development, the longitudinal design to examine the stable influence of attachment on MPD is needed. (2) The self-report measure is used exclusively in the current study. This method, though commonly used, was subject to social desirability. It is suggested that the comprehensive measures should be used, for instance, using the combination of self-report, other-report and other information (e.g., video, official report, monitoring data), in future research.

Second, to better understand the association between attachment and MPD, the multiple-attachment, such as family and peers who both contribute to children’s well-being (Wen and Lin, 2012), should also be investigated simultaneously. Similarly, attachment styles with different figures (father, mother, or other caregiver) may have different effects on the variables considered, it is worth investigating and analyzing separately in future studies. In addition, investigation into the details of mobile phone use could be more comprehensive. In this study, only the frequency of the mobile phone use was investigated and the contents and motivation were not analyzed in depth. In future, the specific functions that might attract more adolescents with insecure attachment and the corresponding drive for MPD are important research issues. For example, the App Timer Mini 2 Pro, which drew on the practice of Chen et al. (2016), can be used to record the duration and content of phone use. This can provide objective evidence to help us better know the mobile phone use of adolescents.

Third, participants’ prior experience in mindfulness practices (or more generally, meditation) had not been assessed. Hence, the possible influence of previous mindfulness experience in the results could not be determined. In future research, previous experience of the individual should be taken into consideration as much as possible. And the mechanism of the protective effect of mindfulness should be studied further in order to provide better guidance for MPD intervention.

## Data Availability

The datasets generated for this study are available on request to the corresponding author.

## Ethics Statement

This study was carried out in accordance with the recommendations of administration committees of the surveyed colleges and universities and from the Human Research Ethics Committee of Shenzhen University with written informed consent from all subjects and their parents. All subjects and their parents gave written informed consent in accordance with the Declaration of Helsinki. The protocol was approved by the Human Research Ethics Committee of Shenzhen University.

## Author Contributions

XL developed the concepts for the study. CH collected and analyzed the data under the guidance of XL. CH and XL wrote the manuscript. Both the authors contributed to the manuscript and approved the final version of the manuscript for submission.

## Conflict of Interest Statement

The authors declare that the research was conducted in the absence of any commercial or financial relationships that could be construed as a potential conflict of interest.
